# Comparison of Twin Block appliance and Herbst appliance in the treatment of Class II malocclusion among children: a meta-analysis

**DOI:** 10.1186/s12903-024-04027-w

**Published:** 2024-02-26

**Authors:** Feifei Xu, Ying Fang, Xiaoling Sui, Yapeng Yao

**Affiliations:** 1 Department of Pediatric Stomatology, Jinan Stomatology Hospital, 101 Jingliu Road, Shizhong District, Jinan City, Shandong Province 250000 P. R. China; 2 Department of Pediatric Dentistry, Affiliated Stomatology Hospital of Guangzhou Medical University, Guangdong Engineering Research Center of Oral Restoration and Reconstruction, Guangzhou Key Laboratory of Basic and Applied Research of Oral Regenerative Medicine, Guangdong, Guangzhou 510182 P. R. China; 3 Department of Pediatric Stomatology, Yantai Stomatological Hospital, Yantai, Shandong 264000 P. R. China

**Keywords:** Twin Block, Herbst, Class II malocclusion, Meta-analysis

## Abstract

**Background:**

Our meta-analysis aimed to evaluate the efficacy of applying Herbst and Twin Block appliances in the treatment of Class II malocclusion among children.

**Methods:**

Databases, including PubMed, Embase, Cochrane Library, Web of Science, China National Knowledge Infrastructure (CNKI), China VIP Database (VIP), and Wanfang were thoroughly searched from inception to August 9, 2023. The outcomes included skeletal, dental, and soft tissue changes. The weighted mean difference (WMD) was used as the effect indicator, and the effect size was expressed with a 95% confidence interval (CI). The heterogeneity of each outcome effect size was tested, and the heterogeneity statistic I^2^ ≥ 50% was analyzed by the random-effect model, otherwise, the fixed-effect model was conducted. Sensitivity analysis was performed.

**Results:**

A total of 12 studies involving 574 patients were included in this meta-analysis. Herbst appliance had a statistically significant increase in mandibular body length (WMD: 1.44, 95% CI: 0.93 to 1.96, *P* < 0.001) compared with the Twin Block appliance. More increases in angle and distance of L1 to mandibular plane (MP) were found in the Herbst appliance compared with the Twin Block appliance. Significant and greater improvements in molar relationship (WMD: 0.79, 95% CI: 0.28 to 1.29, *P* = 0.002), posterior facial height (WMD: -1.23, 95% CI: -2.08 to -0.38, *P* = 0.005), convexity angle (WMD: -1.89, 95% CI: -3.12 to -0.66, *P* = 0.003), and Sella-Nasion plane angle (U1 to SN) (WMD: 3.34, 95% CI: 2.25 to 4.43, *P* < 0.001) were achieved in the Twin Block appliance. Herbst and Twin Block appliances produced similar effects in the skeletal and dentoalveolar changes including Sella-Nasion-point A (SNA), Sella-Nasion-point B, point A-Nasion-point B (ANB), overjet, and overbite.

**Conclusion:**

As the findings revealed both Herbst and Twin Block appliances contributed successfully to the correction of Class II malocclusion. Compared with the Twin Block appliance, the Herbst appliance may have more advantages in mandibular bone movement. Twin Block therapy resulted in more improvement in the aesthetics of the face.

**Supplementary Information:**

The online version contains supplementary material available at 10.1186/s12903-024-04027-w.

## Background

Class II malocclusion, a complex orthodontic issue, is characterized by a convex facial profile due to maxillary retrusion, mandibular protrusion, or both [1]. Approximately one in three individuals suffer from Class II malocclusion [2]. Malocclusion can significantly impact a person's life, affecting oral-facial functions like chewing, speaking, and swallowing, and also has a psychological and social influence due to its effect on appearance [3–5]. The severity of malocclusion can be linked to the progression of the condition, which may worsen as one ages [6]. Hence, implementing an effective treatment strategy for Class II malocclusion is crucial to halt its progression.

Functional appliances, available as both removable and fixed types, are a widely accepted treatment option for correcting Class II malocclusions in growing children [7, 8]. The Herbst appliance, a fixed and rigid functional device, is commonly utilized in treating Class II malocclusion [9]. Using the Herbst appliance allows for effective treatment within a brief period of six to eight months, offering flexibility in choosing the treatment duration during the pubertal growth phase [10]. A previous study reported that treatment with the Herbst appliance stimulates growth in the condylar head and anterior remodeling of the glenoid fossa, leading to an enhanced maxilla-mandibular relationship in patients with growing skeletal Class II issues [9]. The Twin Block is the most favored and extensively utilized removable functional appliance for correcting Class II malocclusion in patients who are still growing [11, 12]. A retrospective analysis indicated the effectiveness of the Twin Block appliance in treating Class II malocclusion, attributable to a mix of skeletal and dentoalveolar alterations in both dental arches [11]. Moreover, the Twin Block enhances facial aesthetics in Class II malocclusion through a blend of alterations in both skeletal and dentoalveolar structures [13]. A randomized controlled trial (RCT) showed that using Herbst for treatment led to a more effective and consistent reduction in overjet compared to the Twin-block [14]. Baysal and Uysal's research indicated that, in comparison to the Herbst appliance, the Twin Block appliance produces a more significant quantitative change in the soft tissue profile [15]. Nevertheless, a study assessing perceived changes in soft tissue profiles using Herbst and Twin Block appliances in Class II malocclusion patients found no noticeable difference in profile improvement between the two appliances [16]. In view of the conflicting results, and no consensus has been reached in the literature regarding which type of appliance is more effective in the treatment of patients with this malocclusion, a meta-analysis is necessary.

This meta-analysis was undertaken to evaluate the efficacy of applying Herbst and Twin Block appliances in the treatment of Class II malocclusion among children by comparing radiographic cephalometric in skeletal, dental, and soft tissue changes to guide clinicians in their choice.

## Methods

This study adhered to the guidelines of the Preferred Reporting Items for Systemic Review and Meta-Analysis (PRISMA) statement [17].

### Search strategy

From inception until August 9, 2023, a search for relevant studies was conducted across multiple electronic databases, including PubMed, Embase, Cochrane Library, Web of Science, China National Knowledge Infrastructure (CNKI), China VIP Database (VIP), and Wanfang data. The search strategy included the combination of keywords “Functional Orthodontic Appliances”, “Herbst”, “Twin Block”, “unilateral posterior crossbite”, and “Class II malocclusion”. The search terms and strategy for PubMed are in Supplementary File 1.

### Study inclusion and exclusion criteria

Inclusion criteria were conducted according to the PICOS principles: (1) P (participants): children with Class II malocclusion; (2) I (intervention): Herbst appliance; (3) C (comparison): Twin block appliance; (4) O (outcome): cephalometric changes of bones [Sella-Nasion-point A (SNA), Sella-Nasion-point B (SNB), point A-Nasion-point B (ANB), mandibular body length (Go-Gn), effective mandibular length (Co-Gn), overjet, overbite, interincisal angle, molar relationship, Nasion-submental point (N-Me), anterior nasal spine-submental point (ANS-Me), Sella-Gonion (S-Go), ramus length (Co-Go)], teeth [Sella-Nasion plane angle (U1 to SN), the distance from the long axis of the maxillary incisor to the palatal plane (U1 to palatal plane), the distance between long axis of maxillary molar and palatal plane (U6 to palatal plane), angle and distance between the axis of mandibular incisor and the mandibular plane (L1 to MP), and distance from long axis of mandibular first molar to mandibular plane (L6 to MP], and soft tissue changes [nasolabial angle, hyperdivergent mandibular growth (SN-GoGn), convexity angle (N-Sn-Pog), the total facial convexity angle (N-Prn-Pog), and lower facial height ratio]; (5) S (study design): cohort studies and RCTs; (6) study published in English and Chinese.

Exclusion criteria were: (1) animal experiments and models; (2) conference abstracts, case reports, meta-analyses, reviews, editorials, letters, protocols, errata, and guidelines; (3) studies that are inconsistent with the topic of our study; (4) trial registration records.

### Methodological quality assessments and data extraction

To evaluate the quality of RCTs, the modified Jadad scale [18] was employed, focusing on four criteria: 1) methods of generating random series (0–2 points); 2) randomization concealment (0–2 points); 3) blinding methodology (0–2 points); 4) analysis of withdrawals (0–1 points). RCTs scoring 1–3 were classified as low quality, whereas those with a score of 4–7 were considered high quality. The evaluation of the quality of cohort studies was conducted using the modified Newcastle–Ottawa Scale (NOS) [19], consisting of nine points assessing selection, comparability, and exposure or outcome. The scale allocates up to four stars for selection, two for comparability, and three for exposure or outcome. With a total of 9 points, scores of 0–3 were deemed low quality, 4–6 medium quality, and 7–9 high quality.

The data were extracted from included studies containing the first author’s name, publication year, study design, treatment types, sample size, age of the included population, gender, treatment duration, SNA, SNB, ANB, outcomes, and methodological quality assessment scores. Data extraction was performed by two authors (Feifei Xu and Ying Fan) independently. When disputes arise, the decision is made in consultation with the third author (Xiaoling Sui).

### Outcomes definition

SNA angle referred to the anteroposterior position of the maxilla in relation to the skull base. SNB angle was defined as the anteroposterior position of the mandible in relation to the skull base. ANB was the relation between the maxilla and the mandible. Overjet was a horizontal distance between incisors edges. Overbite was a vertical distance between the incisors edges. The interincisal angle was the angle between the long axis of the most prominent (anteriorly positioned) maxillary and mandibular first incisors. Molar relationship was measured as the distance between the projections of the mesial contact points of the upper and lower first permanent molars on the functional occlusal plane. N-Me was a distance from the Nasion to the submental point. ANS-Me was the distance from the anterior nasal spine to the submental point. S-Go was the linear distance between the Sella and Gonion landmarks.

U1 to SN referred to the angle between the long axis of the upper central incisor and the SN plane. SN-GoGn was defined as the inclination of the mandibular plane in relation to the anterior cranial base. N-Sn-Pog was the angle subtended by nasion, subnasale, and soft tissue pogonion. N-Prn-Pog was defined as the angle subtended by nasion, pronasale, and soft tissue pogonion. Lower facial height ratio referred to the Subnasale-stomion/stomion-gnathion.

### Statistical analysis

All studies were analyzed using Stata 15.1 software (Stata Corporation, College Station, TX, USA). The effect indicator was represented by the weighted mean difference (WMD) and a 95% confidence interval (CI). For each outcome, heterogeneity was assessed using I^2^ statistic. When conducting meta-analyses, the I^2^ statistic is a tool used to measure heterogeneity between studies. Inter-study heterogeneity refers to the degree of difference between the results of different studies, which can help us determine whether it is appropriate to combine these studies together for meta-analysis. The I^2^ statistic typically ranges from 0 to 100%. It represents the percentage of heterogeneity between studies. When the I^2^ statistic was 50% or greater, a random-effect model was used for analysis; otherwise, a fixed-effect model was applied.

An analysis to assess sensitivity was conducted to ascertain if omitting specific studies would markedly influence the overall results. This involved recalculating the aggregate effect size with each study removed individually, then comparing these recalculations with the original meta-analysis. This comparison aimed to evaluate the impact of the omitted studies on both the cumulative effect size and the meta-analysis’s consistency. A minor or non-existent change in outcome post-exclusion indicated low sensitivity, suggesting that the findings were more solid and trustworthy. Conversely, a significant alteration or a completely contradictory outcome post-exclusion indicated high sensitivity, pointing to a lower reliability of the results. A *P* < 0.05 was considered statistically significant.

## Results

### Study selection and characteristics of included studies

**Fig. 1:**
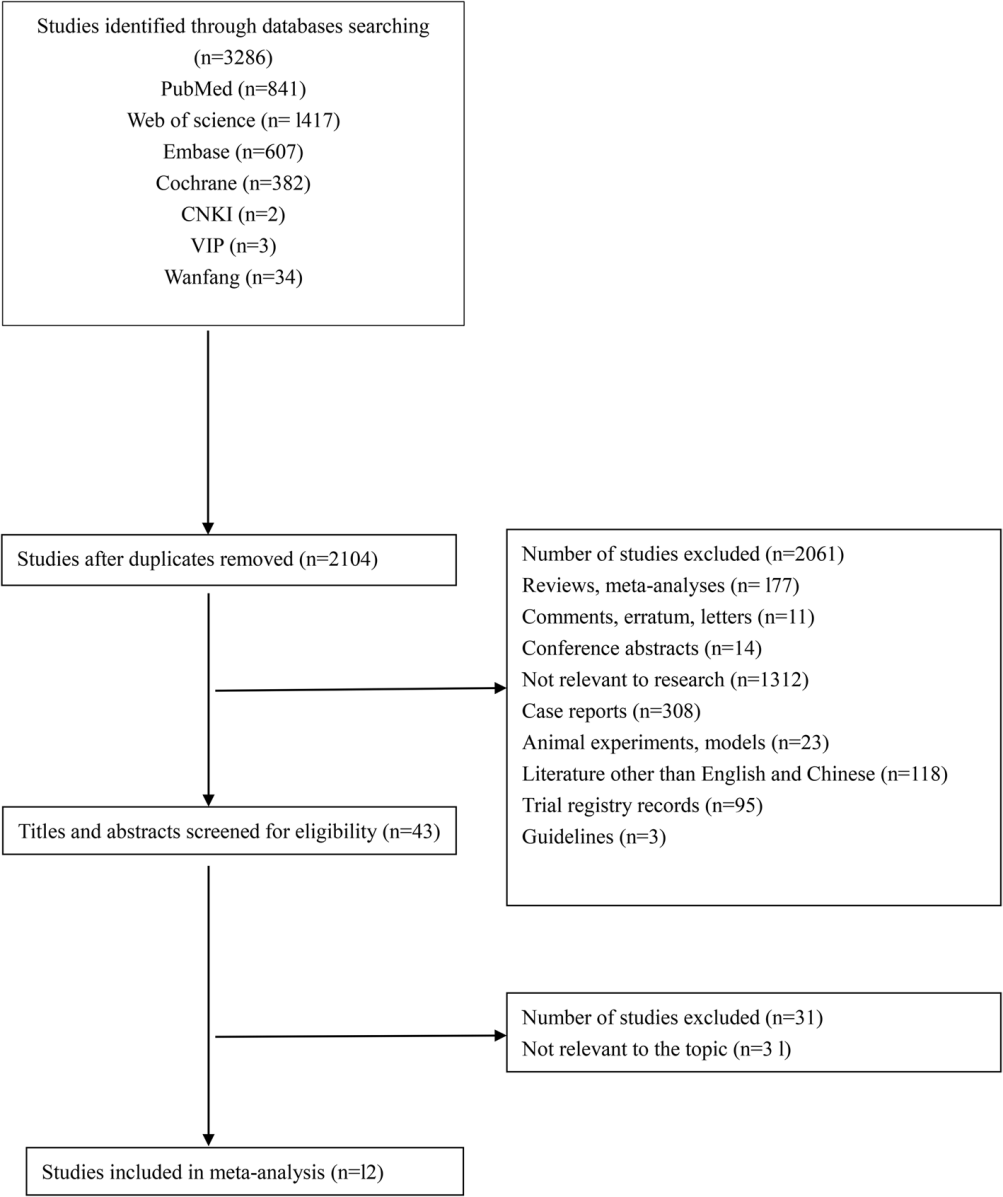
The flowchart of the literature selection

A total of 3,286 studies were located via database searches, comprising 841 from PubMed, 1,417 from Web of Science, 607 from Embase, 382 from Cochrane, 2 from CNKI, 3 from VIP, and 34 from Wanfang. Once duplicates were eliminated, the count reduced to 2,104 studies. Subsequently, 43 titles and abstracts underwent screening according to specific inclusion and exclusion criteria. Finally, 12 studies [15, 16, 20–29] were included in the study. Figure 1 presents a summary of the literature selection process. Of the selected studies, 8 were cohort studies, and 4 were RCTs. Within the selected research, 5 studies were classified as high quality, 6 as medium quality, and 1 as low quality. The studies collectively involved 574 patients, with 290 undergoing treatment with the Twin-block appliance and 284 with the Herbst appliance. Detailed information about these studies is provided in Table 1.

**Table 1:** Basic characteristics of included studies

Author	Year	Study design	Treatment	N	Age	Male/female (N)	Treatment time	SNA(°)	SNB(°)	ANB(°)	Outcomes	NOS quality assessment	Jadad quality assessment
Schaefer	2004	Prospective cohort	Stainless steel Crown Herbst appliance	28	11 years 9 months ± 1 year	7/21	13 months	81.7 ± 2.8	76.2 ± 2.4	5.5 ± 1.8	Skeletal, dentoalveolar, soft tissue	6	
			Twin block appliance	28	12 years 5 months ± 1 year	7/21		82.0 ± 3.3	75.8 ± 2.7	6.2 ± 1.7			
Baysal	2013	RCT	Herbst appliance	20	12.74 ± 1.43 years	9/11	15.81 ± 5.96 months	80.92 ± 1.13	74.1 ± 2.08	6.77 ± 1.56	Skeletal, dentoalveolar, soft tissue		4
			Twin block appliance	20	13 ± 1.32 years	10/10	16.20 ± 7.54 months	80.72 ± 0.99	74.7 ± 1.77	6.02 ± 1.17			
Baysal	2014	RCT	Herbst appliance	20	12.74 ± 1.43 years	9/11	15.81 ± 5.96 months	80.92 ± 1.13	74.1 ± 2.08	6.77 ± 1.56	Skeletal, dentoalveolar		4
			Twin block appliance	20	13 ± 1.32 years	10/10	16.20 ± 7.54 months	80.72 ± 0.99	74.7 ± 1.77	6.02 ± 1.17			
Seker	2020	Retrospective cohort	Stainless steel Crown Herbst appliance	20	12.58 ± 0.5 years	5/15	9.2 ± 2 months				Dentoalveolar	5	
			Twin block appliance	20	11.08 ± 0.3 years	7/13	11.1 ± 4 months						
Guler	2020	RCT	Herbst appliance	20	13.11 ± 2.22 years	8/12	9.15 ± 0.93 months				Soft tissue		3
			Twin block appliance	20	12.35 ± 1.01 years	8/12	9.55 ± 1.46 months						
Kannan	2022	Retrospective cohort	banded Herbst	15	male: 13.5 years ± 1 month female: 12.8 years ± 2 months	10/5	6 months ± 1.1 months				Skeletal, soft tissue	5	
			Twin block	15	male: 13.1 years ± 2 months female: 12 years ± 1 month	11/4							
Kurt	2010	Retrospective cohort	Herbst appliance	10	14.56 ± 1.59 years	4/6		79.9 ± 3.47	73.85 ± 2.82	6.05 ± 1.16	Skeletal, dentoalveolar, soft tissue	5	
			Twin block appliance	10	12.91 ± 0.90 years	4/6		78.8 ± 4.09	73.45 ± 3.13	5.35 ± 1.87			
O'Brien	2003	RCT	Herbst appliance	105	12.74 (95% CI 12.48–12.99) years	50/55	5.81 (95% CI 5.13–6.48) months				Skeletal, dentoalveolar		4
			Twin block appliance	110	12.41 (95% CI 12.17–12.63) years	48/62	11.22 (95% CI 9.58–12.86) months						
Yanqi Wu	2023	Retrospective cohort	Herbst appliance	11	11.55 ± 0.69 years	4/7	10.18 ± 3.06 months	81.02 ± 2.13	73.66 ± 3.37	7.70 ± 3.70	Skeletal, dentoalveolar, soft tissue	5	
			Twin block appliance	12	11.00 ± 1.04 years	7/5	10.16 ± 5.46 months	81.71 ± 2.40	74.08 ± 2.34	7.80 ± 1.40			
Yu Song	2008	Retrospective cohort	Herbst appliance	20	11.6 (10, 13) years						Skeletal, dentoalveolar	5	
			Twin block appliance	20									
Ganfang Deng	2013	Retrospective cohort	Herbst appliance	16	10 (8, 14) years	18/14					Skeletal, dentoalveolar	7	
			Twin block appliance	16									
Zhen Yang	2011	Retrospective cohort	Herbst appliance	19	10.5 ± 0.6 years	11/8					Skeletal, dentoalveolar	7	
			Twin block appliance	19	10.3 ± 0.5 years	12/7							

*SNA* Sella-Nasion-point A, *SNB* Sella-Nasion-point B, *ANB* point A-Nasion-point B, *NOS* Newcastle–Ottawa Scale, *RCT* randomized controlled trial, *CI* confidence interval

### Maxillary skeletal changes between patients treated with Herbst and Twin-block appliances

#### SNA change

A total of 5 groups of data from 5 articles were included to assess the SNA change between patients treated with Herbst and Twin-block appliances. The heterogeneity test results showed that I^2^ = 0.0%, so the fixed-effect model was analyzed. The result could not prove a statically difference in SNA change between patients treated with Herbst and Twin-block appliances (WMD: -0.19, 95% CI: -0.48 to 0.11, *P* = 0.219) (Table 2).

**Table 2:** Comparison of Twin Block appliance and Herbst appliance in the treatment of Class II malocclusion in children

Indicators	Outcomes	WMD (95% CI)	*P*	I^2^
**Maxillary skeletal changes**				
SNA change	Hebst VS Twin Block			
	Overall	-0.19 (-0.48, 0.11)	0.219	0.0
	Sensitivity analysis	-0.19 (-0.48, 0.11)		
**Mandibular skeletal changes**				
SNB change	Hebst VS Twin Block			
	Overall	0.26 (-0.54, 1.07)	0.524	90.5
	Sensitivity analysis	0.26 (-0.54, 1.07)		
Go-Gn change	Hebst VS Twin Block			
	Overall	1.44 (0.93, 1.96)	< 0.001	0.0
	Sensitivity analysis	1.44 (0.93, 1.96)		
Co-Gn change	Hebst VS Twin Block			
	Overall	0.16 (-0.88, 1.19)	0.766	71.7
	Sensitivity analysis	0.16 (-0.88, 1.19)		
**Interdental**				
Overjet change	Hebst VS Twin Block			
	Overall	1.11 (-0.18, 2.41)	0.091	81.2
	Sensitivity analysis	1.11 (-0.18, 2.41)		
Overbite change	Hebst VS Twin Block			
	Overall	0.13 (-0.42, 0.69)	0.639	33.0
	Sensitivity analysis	0.13 (-0.42, 0.69)		
Interincisal angle	Hebst VS Twin Block			
	Overall	-3.59 (-7.66, 0.49)	0.085	0.0
	Sensitivity analysis	-3.59 (-7.66, 0.49)		
Molar relationship change	Hebst VS Twin Block			
	Overall	0.79 (0.28, 1.29)	0.002	0.0
	Sensitivity analysis	0.796 (0.28, 1.29)		
**Maxillary/Mandibular**				
ANB change	Hebst VS Twin Block			
	Overall	-0.48 (-1.69, 0.74)	0.442	95.8
	Sensitivity analysis	-0.48 (-1.69, 0.74)		
**Vertical skeletal**				
N-Me change	Hebst VS Twin Block			
	Overall	-0.90 (-1.99, 0.19)	0.106	0.0
	Sensitivity analysis	-0.90 (-1.99, 0.19)		
ANS-Me change	Hebst VS Twin Block			
	Overall	-0.85 (-2.19, 0.49)	0.212	70.4
	Sensitivity analysis	-0.85 (-2.19, 0.49)		
S-Go change	Hebst VS Twin Block			
	Overall	-1.23 (-2.08, -0.38)	0.005	48.3
	Sensitivity analysis	-1.23 (-2.08, -0.38)		
Co-Go change	Hebst VS Twin Block			
	Overall	-0.53 (-1.73, 0.66)	0.380	78.9
	Sensitivity analysis	-0.53 (-1.73, 0.66)		
**Soft tissue**				
Nasolabial angle change	Hebst VS Twin Block			
	Overall	-1.34 (-5.53, 2.85)	0.531	65.3
	Sensitivity analysis	-1.34 (-5.53,2.85)		
SN-GoGn change	Hebst VS Twin Block			
	Overall	0.07 (-0.47, 0.61)	0.793	0.0
	Sensitivity analysis	0.07 (-0.47, 0.61)		
N-Sn-Pog change	Hebst VS Twin Block			
	Overall	-1.89 (-3.12, -0.66)	0.003	0.0
	Sensitivity analysis	-1.89 (-3.12, -0.66)		
N-Prn-Pog change	Hebst VS Twin Block			
	Overall	-0.95 (-1.94, 0.05)	0.062	0.0
	Sensitivity analysis	-0.95 (-1.94, 0.05)		
Lower facial height ratio change	Hebst VS Twin Block			
	Overall	0.03 (-0.03, 0.08)	0.343	0.0
	Sensitivity analysis	0.03 (-0.03, 0.08)		
**Maxillary dentoalveolar**				
U1 to SN change	Hebst VS Twin Block			
	Overall	3.34 (2.25, 4.43)	< 0.001	75.7
	Sensitivity analysis	3.34 (2.25, 4.43)		
U1 to palatal plane change	Hebst VS Twin Block			
	Overall	-0.16 (-1.12, 0.81)	0.751	72.7
	Sensitivity analysis	-0.16 (-1.12, 0.81)		
U6 to palatal plane change	Hebst VS Twin Block			
	Overall	-0.36 (-1.20, 0.48)	0.402	76.6
	Sensitivity analysis	-0.36 (-1.20, 0.48)		
**Mandibular dentoalveolar**				
L1 to MP change (°)	Hebst VS Twin Block			
	Overall	2.64 (2.09, 3.19)	< 0.001	0.0
	Sensitivity analysis	2.64 (2.09, 3.19)		
L1 to MP change (mm)	Hebst VS Twin Block			
	Overall	0.76 (0.22, 1.31)	0.006	0.0
	Sensitivity analysis	0.76 (0.22, 1.31)		
L6 to MP change (mm)	Hebst VS Twin Block			
	Overall	-0.36 (-1.36, 0.64)	0.482	61.8
	Sensitivity analysis	-0.36 (-1.36, 0.64)		

*WMD* weighted mean difference, *CI* confidence interval, *SNA* Sella-Nasion-point A, *SNB* Sella-Nasion-point B, *Go-Gn* mandibular body length, *Co-Gn* effective mandibular length, *N-Me* Nasion-submental point, *ANS-Me* anterior nasal spine-submental point, *S-Go* Sella-Gonion, *Co-Go* ramus length, *ANB* point A-Nasion-point B, *SN-GoGn* hyperdivergent mandibular growth, *N -Sn-Pog* convexity angle, *N-Prn-Pog* total facial convexity angle, *U1 to SN* Sella-Nasion plane angle, *MP* mandibular plane

### Mandibular skeletal changes between patients treated with Herbst and Twin-block appliances

#### SNB change

Totally, 8 studies were included to assess SNB change between patients treated with Herbst and Twin-block appliances. The random-effect model results showed that there was no significant difference in SNB changes between patients treated with Herbst and Twin-block appliances (I^2^ = 90.5%, WMD: 0.26, 95% CI: -0.54 to 1.07, *P* = 0.524) (Table 2).

#### Go-Gn change

**Fig. 2:**
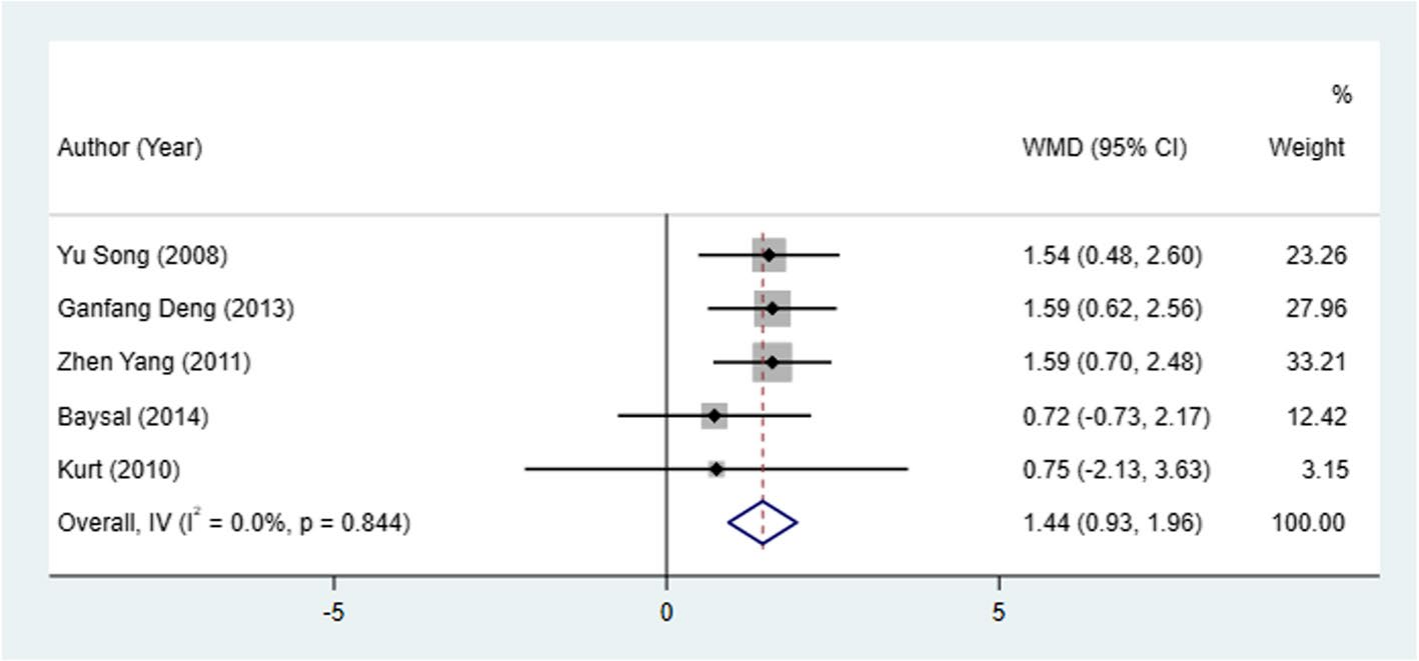
Go-Gn change between patients treated with Herbst and Twin-block appliances

Go-Gn change between patients treated with Herbst and Twin-block appliances was investigated in 5 studies. The result from the fixed-effect model analysis indicated that the Herbst appliance had more Go-Gn change than the Twin-block appliance (I^2^ = 0.0%, WMD: 1.44, 95% CI: 0.93 to 1.96, *P* < 0.001) (Table 2, Fig. 2).

#### Co-Gn change

Six studies evaluated Co-Gn change between patients treated with Herbst and Twin-block appliances. There was no significant difference in Co-Gn change between Twin-block appliance and Herbst appliance (I^2^ = 71.7%, WMD: 0.156, 95% CI: -0.88 to 1.19, *P* = 0.766) (Table 2).

### Interdental skeletal changes between patients treated with Herbst and Twin-block appliances

#### Overjet, overbite, and interincisal angle changes

Overjet, overbite, and interincisal angle changes were analyzed in 5, 3, and 2 studies, respectively. No significant differences in overjet (I^2^ = 81.2%, WMD: 1.11, 95% CI: -0.18 to 2.41, *P* = 0.091), overbite (I^2^ = 33.0%, WMD: 0.13, 95% CI: -0.42 to 0.69), *P* = 0.639), and interincisal angle (I^2^ = 0.0%, WMD: -3.59, 95% CI: -7.66 to 0.49, *P* = 0.085) changes between Twin-block appliance and Herbst appliance were observed (Table 2).

#### Molar relationship change

**Fig. 3:**
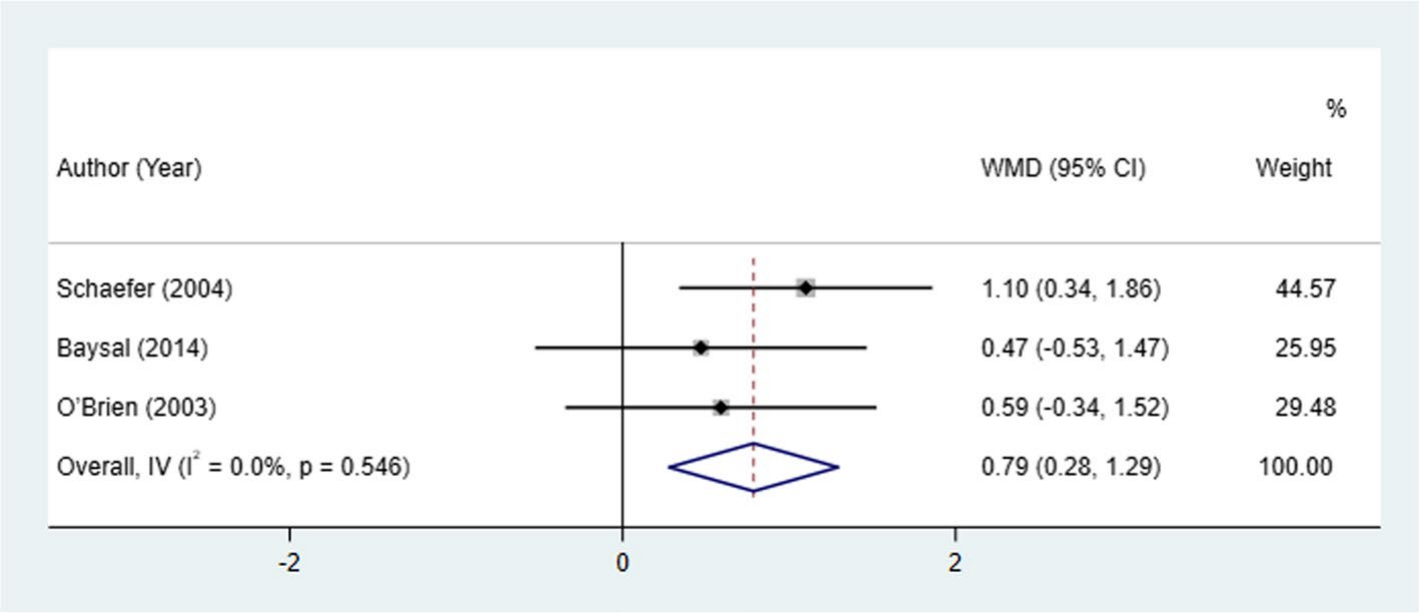
Molar relationship changes between patients treated with Herbst and Twin-block appliances

Molar relationship changes between patients treated with Herbst and Twin-block appliances were examined in 3 studies. The fixed-effect model revealed a significant increase in molar relationship in the Herbst appliance compared with the Twin-block appliance (I^2^ = 0.0% WMD: 0.79, 95% CI: 0.28 to 1.29, *P* = 0.002), indicating greater correction in molar relationship was found in the Twin-block appliance compared with Herbst appliance (Table 2, Fig. 3).

### Maxillary/Mandibular changes between patients treated with Herbst and Twin-block appliances

#### ANB angle change

Change in ANB angle between patients treated with Herbst and Twin-block appliances was calculated in 8 studies. No difference in ANB angle change between Herbst and Twin-block appliances was shown (I^2^ = 95.8%, WMD: -0.48, 95% CI: -1.69 to 0.74, *P* = 0.442) (Table 2).

### Vertical skeletal changes between patients treated with Herbst and Twin-block appliances

#### N-Me, ANS-Me, and Co-Go changes

N-Me, ANS-Me, and Co-Go changes between patients treated with Herbst and Twin-block appliances were investigated in 2, 4, and 6 studies, respectively. There were no significant differences in N-Me change, ANS-Me change, and Co-Go change between patients treated with Herbst and Twin-block appliances (Table 2).

#### S-Go change

**Fig. 4:**
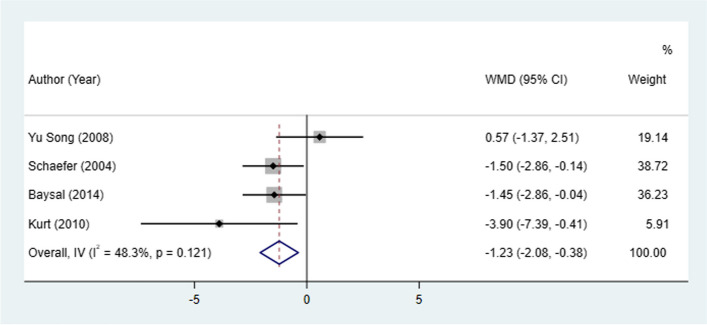
S-Go change between patients treated with Herbst and Twin-block appliances

Four studies assessed S-Go change between patients treated with Herbst and Twin-block appliances. The Herbst appliance had less S-Go change compared with the Twin-block appliance (I^2^ = 48.3%, WMD: -1.23, 95% CI: -2.08 to -0.38, *P* = 0.005) (Table 2, Fig. 4).

### Soft tissue changes between patients treated with Herbst and Twin-block appliances

#### Nasolabial angle, SN-GoGn, N-Prn-Pog, and lower facial height ratio changes

Three studies, five studies, two studies, and two studies respectively evaluated nasolabial angle, SN-GoGn, N-Prn-Pog, and lower facial height ratio changes between patients treated with Herbst and Twin-block appliances. There were no significant differences between patients treated with Herbst and Twin-block appliances in nasolabial angle (I^2^ = 65.3%, WMD: -1.34, 95% CI: -5.53 to 2.85, *P* = 0.531), SN-GoGn (I^2^ = 0.0%, WMD: 0.07, 95% CI: -0.47 to 0.61, *P* = 0.793), N-Prn-Pog (I^2^ = 0.0%, WMD: -0.95, 95% CI: -1.94 to 0.05, *P* = 0.062), and lower facial height ratio (I^2^ = 0.0%, WMD: 0.03, 95% CI: -0.03 to 0.08, *P* = 0.343) changes (Table 2).

#### N-Sn-Pog change

**Fig. 5:**
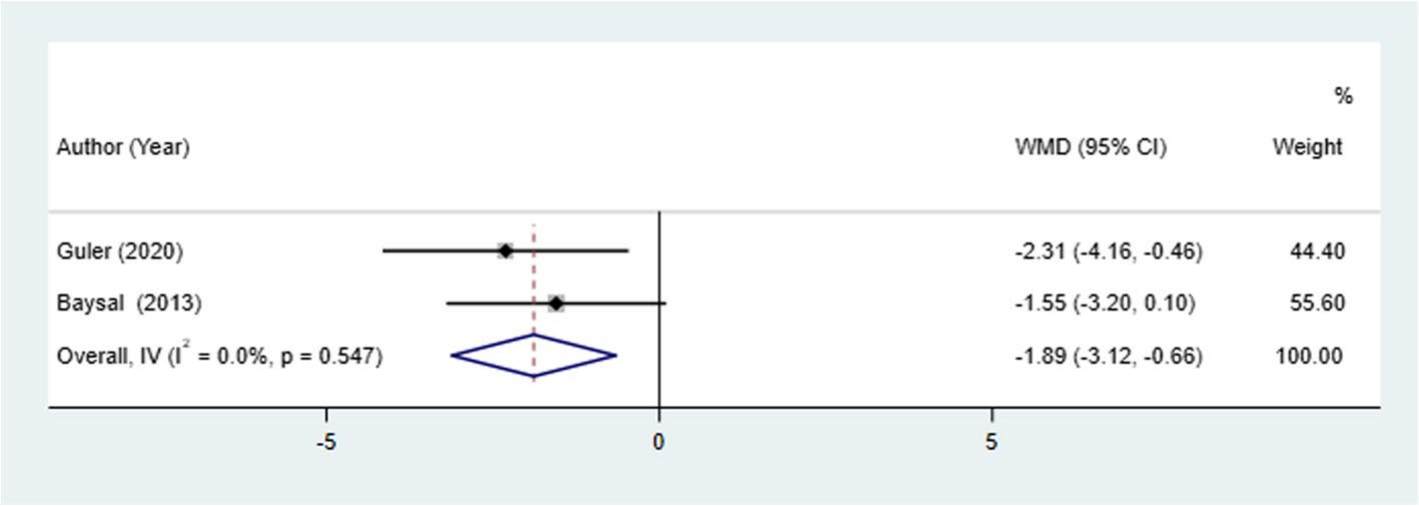
N-Sn-Pog change between patients treated with Herbst and Twin-block appliances

N-Sn-Pog change between patients treated with Herbst and Twin-block appliances was examined in 2 studies. The result from the fixed-effect model demonstrated that a lower N-Sn-Pog change was observed in the Herbst appliance compared with the Twin-block appliance (I^2^ = 0.0%, WMD: -1.89, 95% CI: -3.12 to -0.66, *P* = 0.003) (Table 2, Fig. 5).

### Maxillary dentoalveolar changes between patients treated with Herbst and Twin-block appliances

#### U1 to SN change

**Fig. 6:**
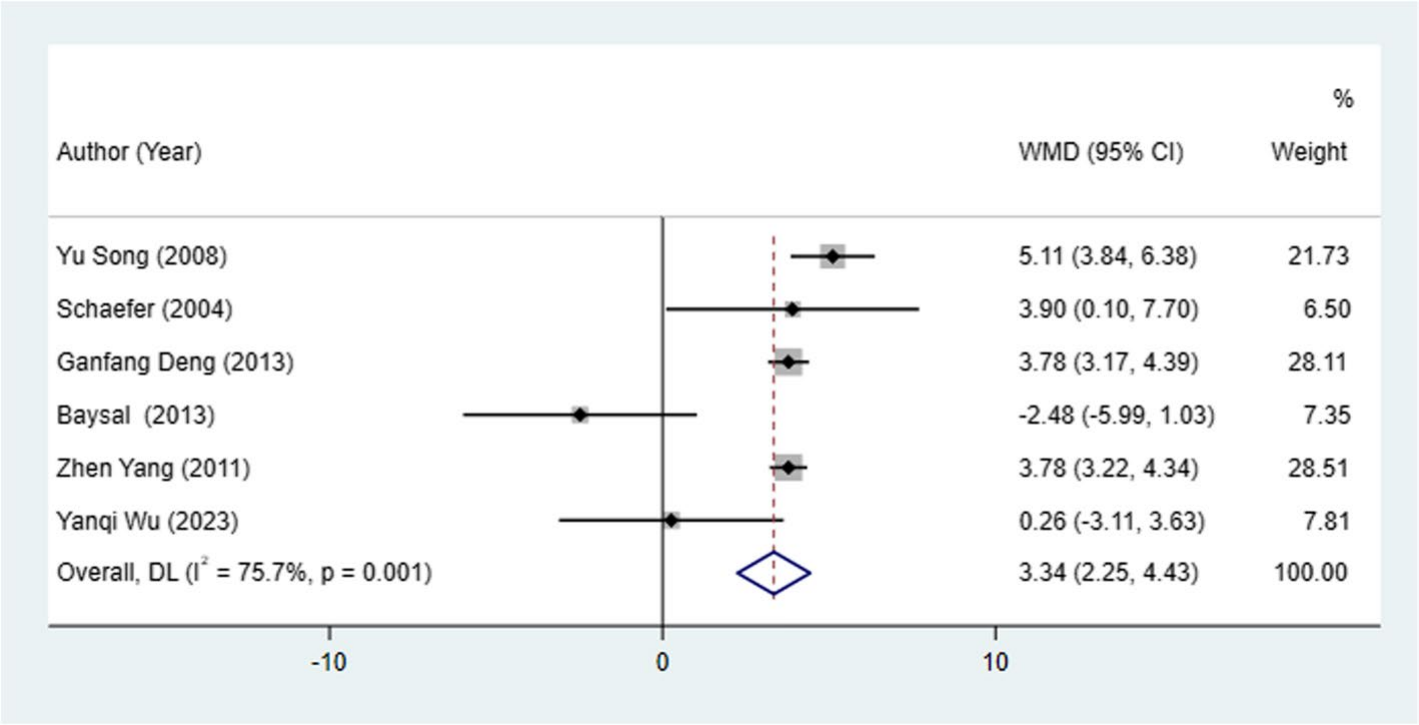
U1 to SN change between patients treated with Herbst and Twin-block appliances

Five studies assessed U1 to SN change between patients treated with the Herbst and Twin-block appliances. The result from the random-effect model analysis showed that Herbst appliance had an increase in U1 to SN compared with the Twin-block appliance (I^2^ = 75.7%, WMD: 3.34, 95% CI: 2.25 to 4.43, *P* < 0.001), suggesting that Twin-block appliance had more improvement of U1 to SN than the Herbst appliance (Table 2, Fig. 6).

#### U1 to palatal plane change and U6 to palatal plane change

U1 to palatal plane and U6 to palatal plane changes were investigated in 4, and 3 studies, respectively. No differences in U1 to palatal plane and U6 to palatal plane change between patients treated with Herbst and Twin-block appliances were observed (Table 2).

### Mandibular dentoalveolar changes between patients treated with Herbst and Twin-block appliances

#### L1 to MP angle change and L1 to MP change

**Fig. 7:**
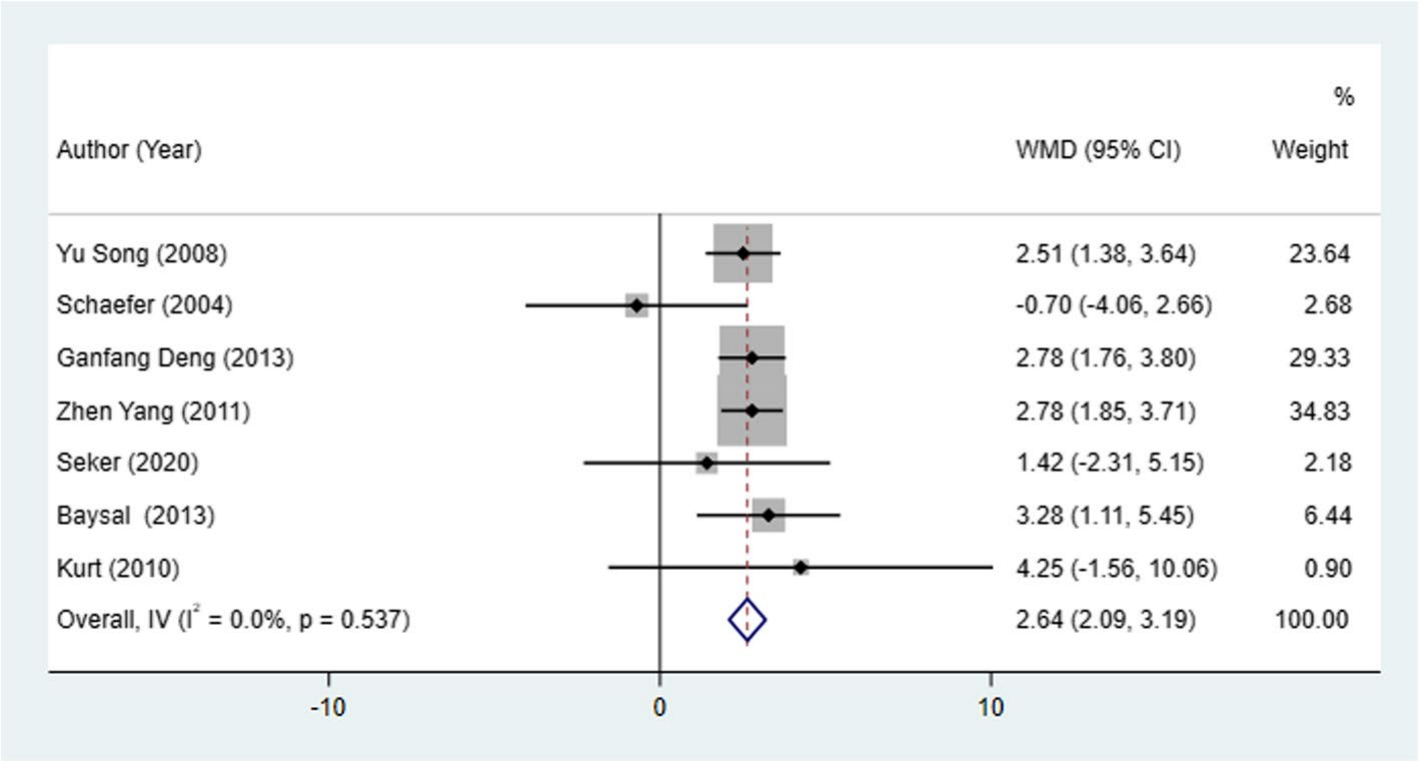
L1 to MP angle change between patients treated with Herbst and Twin-block appliances

**Fig. 8:**
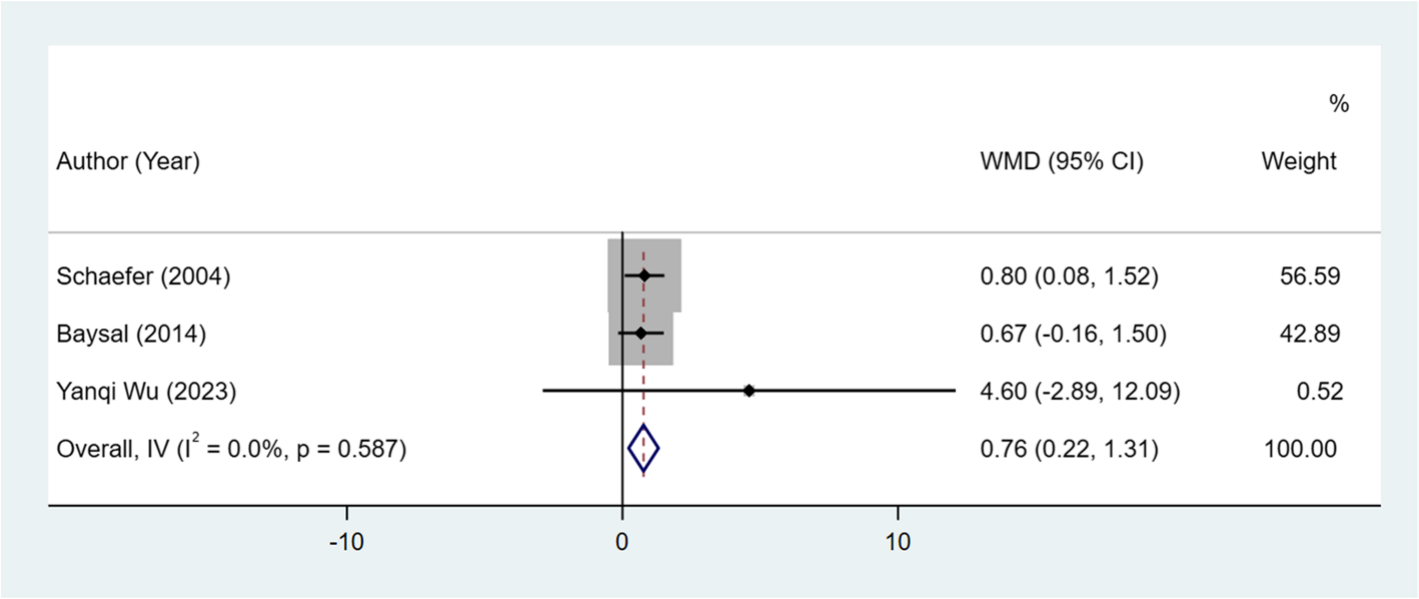
L1 to MP change between patients treated with Herbst and Twin-block appliances

Seven studies and 3 studies, respectively, examined L1 to MP angle change and L1 to MP change between patients treated with Herbst and Twin-block appliances. The Herbst appliance had significant increases in the L1 to MP angle (I^2^ = 0.0%, WMD: 2.64, 95% CI: 2.09 to 3.19, *P* < 0.001) (Table 2, Fig. 7) and L1 to MP (I^2^ = 0.0%, WMD: 0.76, 95% CI: 0.22 to 1.31, *P* = 0.006) than the Twin-block appliance (Table 2, Fig. 8).

#### L6 to MP change

L6 to MP change between patients treated with Herbst and Twin-block appliances was assessed in 2 studies. The heterogeneity test results showed that I^2^ = 61.8%, thereby, the random-effect model was used. No significant difference was found between patients treated with Herbst and Twin-block appliances in L6 to MP change (WMD: -0.36, 95% CI: -1.36 to 0.64, *P* = 0.482) (Table 2).

## Discussion

In orthodontics, Class II malocclusions are the most frequently encountered cases, and the Herbst and Twin-block appliances have emerged as the leading and most extensively adopted functional appliances for treating Class II malocclusion in young patients [11, 16]. However, there is no consensus in existing research regarding which appliance is superior. This meta-analysis was conducted to determine the efficacy of Herbst and Twin Block appliances in the treatment of Class II malocclusion in children by comparing radiographic cephalometric changes in skeletal, dental, and soft tissue.

Our findings indicated a superior performance of the Herbst appliance in enhancing the length of the mandibular body. More increases in angle and distance of L1 to MP were found in Herbst appliance compared with the Twin Block appliance. The Twin Block appliance demonstrated more substantial enhancements in the molar relationship, posterior facial height, convexity angle, and the U1 to SN measurements.

The Herbst appliance, a fixed dental apparatus, maintains the mandible in an extended forward position, thereby promoting the growth of both the condyle and the mandible [30]. In this meta-analysis, the Herbst appliance was more effective in enhancing the length of the mandibular body. An earlier study showed that in terms of maxillary and mandibular movements, the Herbst appliance demonstrated more favorable changes compared to the Twin Block appliance [31]. A research assessing the dentoskeletal impacts of Herbst and Twin Block appliance treatments on skeletal Class II malocclusion found that the Herbst appliance could be beneficial for patients with skeletal Class II conditions, particularly those exhibiting mandibular dentoalveolar retrusion [21]. We also observed that the Herbst appliance had a more obvious change on L1 to MP. A cephalometric study revealed that the use of a fixed appliance configuration in the mandible while employing the Herbst appliance led to a greater proclination of the mandibular incisors [32]. Despite employing an anchorage consisting of a lingually modified lingual arch for the Herbst appliance, positioned away from the lingual surface of the incisors, and a fixed transpalatal bar in the upper arch, there was a significant proclination observed in the mandibular incisors [33].

This meta-analysis indicated a more substantial improvement in molar alignment among children treated with the Twin-block appliance. Earlier research also found that therapy using the Twin-block appliance led to more pronounced skeletal adjustments for molar correction [21]. In the study comparing Twin Block and Herbst appliances, Schaefer et al. discovered that Twin Block appliances achieved a more significant correction in molar relationships [20]. We found that Twin Block induced statistical changes in U1 to SN. An earlier study noted that while Herbst and Twin-Block appliances showed varying degrees of upper incisor retroclination and lower incisor proclination, the reduction in the U1 to SN angle did not exhibit a notable difference [26]. The comparison effect of Herbst and Twin-Block on U1 to SN needs further elaboration. In orthodontics, attaining pleasing facial aesthetics is crucial, especially since patients with Class II malocclusion frequently exhibit several unfavorable facial traits that can negatively impact their social well-being [34]. This meta-analysis analyzed soft-tissue profile changes after Class II Twin-block treatment and found improvements in posterior facial height, and convexity angle among children receiving the Twin-block appliance. A previous study supported our findings, showing that Twin-block therapy led to a more significant increase in the height of the mandibular ramus, also known as the posterior facial height [20]. Findings from another study determined that the Twin Block exerted a forward and downward force on the mandible, resulting in an increased posterior facial height [11]. In a prospective study assessing soft tissue aesthetic changes, there was a significant reduction in facial convexity and upper lip protrusion following treatment with a modified Twin Block appliance [34]. A retrospective case–control study revealed that the aesthetic assessment yielded notably better outcomes in the Twin Block group, particularly in terms of diminishing facial convexity [35].

Both the appliances produced similar effects in the skeletal and dentoalveolar changes including SNA, SNB, ANB, overjet, and overbite. A previous analysis indicated that patients treated with the Twin Block appliance experienced a substantial decrease in both overjet and overbite compared to those who did not receive any treatment [11]. According to the research conducted by Smailienė et al., using the Twin-block appliance led to an advancement of the mandible, evidenced by an increase of 0.91° in the SNB angle and a decrease of 0.15° in the ANB angle [36]. A meta-analysis focused on assessing the efficacy of the Herbst appliance in treating patients with Class II malocclusion noted substantial alterations in both the SNA angle and the overbite following treatment with the Herbst appliance [37]. The prospective study demonstrated that treatment with the crowned Herbst appliance resulted in notable skeletal transformations, such as an elevation in the SNB angle, a reduction in the ANB angle, and significant dentoalveolar adjustments including the proclination of lower incisors, retroclination of upper incisors, along with reductions in both overjet and overbite [38]. Wu et al.'s study found that both appliances significantly enhanced overjet relationships, with the Herbst and Twin-Block showing improvements of 5.53 mm and 4.73 mm, respectively [26]. These findings illustrate that both Herbst and Twin-Block appliances bring skeletal and dental changes (improvements) and that the choice between the two appliances demands additional evaluation.

Our findings of this study hold significant clinical implications. The results of this study offer clinicians valuable insights into choosing between the Herbst and Twin Block appliances. Considering that the Herbst appliance may have greater advantages in mandibular bone movement, clinicians can make more precise treatment choices based on individual patient characteristics and needs. This facilitates the development of personalized and effective treatment plans. The study suggests that Twin Block therapy may excel in improving facial aesthetics. Therefore, in treatment planning, clinicians must strike a balance between functional correction and aesthetic considerations. This might entail discussions with patients and their families to ensure alignment between their expectations and the chosen treatment method. Understanding the differential effects of these orthodontic appliances can assist clinicians in communicating effectively with patients, explaining potential treatment outcomes, and collaboratively setting treatment goals. This enhances patient engagement and satisfaction.

This meta-analysis stands as the first meta-analysis comparing the treatment effects of the Herbst appliance and the Twin-block appliance based on comprehensive skeletal, dental, and soft tissue changes. Clinicians can decide the type of appliance according to the treatment benefit, and provide help for the comprehensive skeletal, dental, and soft tissue changes in children with Class II malocclusion. The limitations of this meta-analysis should be mentioned. First, the small number of studies included suggests the need for cautious interpretation of the results. Second, the inability to conduct subgroup analyses for patients with mandibular retrusion or discussions for Herbst appliance due to lack of specific type details in some original studies. Third, evaluating publication bias wasn't feasible as most outcomes had fewer than ten articles. Fourth, this study also included retrospective studies. Retrospective studies typically rely on pre-existing data, which may introduce selection bias. Since the study subjects are chosen after the fact and not through random allocation, there might be some degree of bias that could potentially affect the reliability of the study's results. Due to the inherent nature of retrospective studies, it is challenging to establish causation. While associations can be observed, causal relationships cannot be definitively determined. This could impose limitations on the study’s conclusions and policy recommendations. Future research is needed to better understand the relative efficacy and potential adverse effects of both Herbst and Twin Block appliances.

## Conclusion

Our research revealed that treatments using both Herbst and Twin Block appliances effectively corrected Class II malocclusions, leading to improvements in skeletal, dental, and soft tissue structures. The Herbst appliance appeared to have a greater impact on mandibular bone movement compared to the Twin Block appliance. Meanwhile, the use of the Twin Block appliance yielded more significant results in enhancing the molar relationship and facial aesthetics.

### Supplementary Information

**Supplementary Material 1.**

## Data Availability

The datasets used and/or analyzed during the current study are available from the corresponding author on reasonable request.

## Abbreviations

RCT: Randomized controlled trial
CNKI: China National Knowledge Infrastructure
SNA: Sella-Nasion-point A
SNB: Sella-Nasion-point B
ANB: A-Nasion-point B
ANS-Me: Anterior nasal spine-submental point
S-Go: Sella-Gonion
NOS: Newcastle–Ottawa Scale
L1 to MP: L1 to mandibular plane
U1 to SN: Sella-Nasion plane angle
SNA: Sella-Nasion-point A
SNB: Sella-Nasion-point B
ANB: Point A-Nasion-point B
WMD: Weighted mean difference
CI: Confidence interval
