# Supplementary material for: Comparison of Twin Block appliance and Herbst appliance in the treatment of Class II malocclusion among children: a meta-analysis
Source: BMC Oral Health. 2024 Feb 26;24:278. doi: 10.1186/s12903-024-04027-w (PMC10895725; doi:10.1186/s12903-024-04027-w)
Supplement: Supplementary file 1 — Supplementary Material 1. [file 12903_2024_4027_MOESM1_ESM.docx]

**Supplementary File 1 Search formula in PubMed**

| No. | Search items |
| --- | --- |
| #1 | (((((((((((((((((Orthodontic Appliances, Functional[Title/Abstract]) OR (Appliance, Functional Orthodontic[Title/Abstract])) OR (Appliances, Functional Orthodontic[Title/Abstract])) OR (Functional Orthodontic Appliance[Title/Abstract])) OR (Functional Orthodontic Appliances[Title/Abstract])) OR (Orthodontic Appliance, Functional[Title/Abstract])) OR (Herbst Appliance[Title/Abstract])) OR (Appliance, Herbst[Title/Abstract])) OR (Frankel Function Regulator[Title/Abstract])) OR (Function Regulator, Frankel[Title/Abstract])) OR (Regulator, Frankel Function[Title/Abstract])) OR (Bimler Appliance[Title/Abstract])) OR (Appliance, Bimler[Title/Abstract])) OR (Kinetor[Title/Abstract])) OR (Kinetors[Title/Abstract])) |
| #2 | (((((((((((((((((((((((Activator Appliances[Title/Abstract]) OR (Activator Appliance[Title/Abstract])) OR (Appliance, Activator[Title/Abstract])) OR (Appliances, Activator[Title/Abstract])) OR (Orthodontic Appliances, Activator[Title/Abstract])) OR (Activator Orthodontic Appliance[Title/Abstract])) OR (Activator Orthodontic Appliances[Title/Abstract])) OR (Appliance, Activator Orthodontic[Title/Abstract])) OR (Appliances, Activator Orthodontic[Title/Abstract])) OR (Orthodontic Appliance, Activator[Title/Abstract])) OR (Function Activator[Title/Abstract])) OR (Activator, Function[Title/Abstract])) OR (Activators, Function[Title/Abstract])) OR (Function Activators[Title/Abstract])) OR (Jasper Jumper[Title/Abstract])) OR (Jumper, Jasper[Title/Abstract])) OR (Harvold Activator[Title/Abstract])) OR (Activator, Harvold[Title/Abstract])) OR (Andresen Appliance[Title/Abstract])) OR (Appliance, Andresen[Title/Abstract])) OR (Bionator[Title/Abstract])) OR (Bionators[Title/Abstract])) OR (twin block[Title/Abstract]))) |
| #3 | (((((((((((((((((((((((SGTB appliance[Title/Abstract]) OR (MRC appliance[Title/Abstract])) OR (Simões Network 2[Title/Abstract])) OR (Simões Network 3[Title/Abstract])) OR (modified Balters appliance[Title/Abstract])) OR (U-bow activator Type I[Title/Abstract])) OR (Sander Bite Jumping[Title/Abstract])) OR (doppelplatte appliance[Title/Abstract])) OR (Lehmann appliance[Title/Abstract])) OR (Sydney Magnoglide[Title/Abstract])) OR (Functional orthopedic magnetic appliance[Title/Abstract])) OR (Active Vertical Corrector[Title/Abstract])) OR (functional regulator[Title/Abstract])) OR (bass appliance[Title/Abstract])) OR (herren activator[Title/Abstract])) OR (woodside activator[Title/Abstract])) OR (inclined guide plate[Title/Abstract])) OR (vestibular shield[Title/Abstract])) OR (Maxillary plane guide plate[Title/Abstract])) OR (Upper plane plate[Title/Abstract])) OR (maxilla plane plate[Title/Abstract])) OR (inclined bite-plate[Title/Abstract])) OR (maxillary inclined plane[Title/Abstract]))) |
| #4 | (((((((unilateral posterior crossbite[Title/Abstract]) OR (bilateral posterior crossbite[Title/Abstract])) OR (scissor bite[Title/Abstract])) OR (Brodie bite[Title/Abstract])) |
| #5 | (((((((((((((((((((Open Bite[Title/Abstract]) OR (Bite, Open[Title/Abstract])) OR (Nonocclusion[Title/Abstract])) OR (Openbite[Title/Abstract])) OR (Apertognathia[Title/Abstract])) OR (Overbite[Title/Abstract])) OR (Overbites[Title/Abstract])) OR (Deep-Bite[Title/Abstract])) OR (Deep-Bites[Title/Abstract])) OR (Deep Bite[Title/Abstract])) OR (Deep Bites[Title/Abstract])) OR (Over Bite[Title/Abstract])) OR (Over Bites[Title/Abstract])) OR (Overjet, Dental[Title/Abstract])) OR (Dental Overjet[Title/Abstract])) OR (Dental Overjets[Title/Abstract])) OR (Incisor Protrusion[Title/Abstract])) OR (Incisor Protrusions[Title/Abstract])) OR (Protrusion, Incisor[Title/Abstract]))) |
| #6 | ((((((((((((((((((Malocclusion, Angle Class I[Title/Abstract]) OR (Angle Class I[Title/Abstract])) OR (Class I, Angle[Title/Abstract])) OR (Malocclusion, Angle Class II[Title/Abstract])) OR (Angle Class II[Title/Abstract])) OR (Class II, Angle[Title/Abstract])) OR (Malocclusion, Angle Class II, Division 1[Title/Abstract])) OR (Angle Class II, Division 1[Title/Abstract])) OR (Class II Malocclusion, Division 1[Title/Abstract])) OR (Malocclusion, Angle Class II, Division 2[Title/Abstract])) OR (Class II Malocclusion, Division 2[Title/Abstract])) OR (Angle Class II, Division 2[Title/Abstract])) OR (Malocclusion, Angle Class III[Title/Abstract])) OR (Habsburg Jaw[Title/Abstract])) OR (Prognathism, Mandibular[Title/Abstract])) OR (Hapsburg Jaw[Title/Abstract])) OR (Angle Class III[Title/Abstract])) OR (Underbite[Title/Abstract]))) OR (((((((((((((((Malocclusion[Title/Abstract]) OR (Malocclusions[Title/Abstract])) OR (Tooth Crowding[Title/Abstract])) OR (Crowding, Tooth[Title/Abstract])) OR (Crowdings, Tooth[Title/Abstract])) OR (Crossbite[Title/Abstract])) OR (Crossbites[Title/Abstract])) OR (Cross Bite[Title/Abstract])) OR (Bite, Cross[Title/Abstract])) OR (Bites, Cross[Title/Abstract])) OR (Cross Bites[Title/Abstract])) OR (Angle's Classification[Title/Abstract])) OR (Angle Classification[Title/Abstract])) OR (Angles Classification[Title/Abstract])) OR (Classification, Angle's[Title/Abstract]))) |
| #7 | #1 OR #2 OR #3 |
| #8 | #4 OR #5 OR #6 |
| #9 | #7 AND #8 |
